# Targeting CD24 as a novel immunotherapy for solid cancers

**DOI:** 10.1186/s12964-023-01315-w

**Published:** 2023-11-02

**Authors:** Yan Yang, Guangming Zhu, Li Yang, Yun Yang

**Affiliations:** 1 Xinxiang Engineering Technology Research Center of Tumor-Targeted Drug Development, School of Basic Medical Sciences, Xinxiang Medical University, Xinxiang, 453000 Henan China; 2 Clinical Laboratory, The First People’s Hospital of Taian, Taian 271000, Shandong, China; 3 Department of Obstetrics and Gynecology, The Third Affiliated Hospital of Zhengzhou University, Zhengzhou Key Laboratory of Endometrial Disease Prevention and Treatment Zhengzhou China, Zhengzhou, 450052 Henan China

**Keywords:** CD24, Solid cancer, Cancer Stem Cells, Immunotherapy, Immune checkpoint inhibitors, Cell signaling

## Abstract

Cluster of differentiation 24 (CD24), a mucin-like highly glycosylated molecule has been extensively studied as a cancer stem cell marker in a variety of solid cancers. The functional role of CD24 is either fulfilled by combining with ligands or participating in signal transduction, which mediate the initiation and progression of neoplasms. Recently, CD24 was also described as an innate immune checkpoint with apparent significance in several types of solid cancers. Herein, we review the current understanding of the molecular fundamentals of CD24, the role of CD24 in tumorigenesis and cancer progression, the possibility as a promising therapeutic target and summarized different therapeutic agents or strategies targeting CD24 in solid cancers.

Video Abstract

**Supplementary Information:**

The online version contains supplementary material available at 10.1186/s12964-023-01315-w.

## Introduction

Globally, the incidence of various human cancers is increasing and is still a major cause of mortality. According to GLOBOCAN 2020, an estimated 19,292,789 new cases and 9,958,133 deaths were reported globally produced by the International Agency for Research on Cancer (IARC) (https://gco.iarc.fr/)[1] . Therefore, there is a growing need for the development of new therapeutic regimen that can improve the survival of cancer patients.

Over the last decade, a new understanding of tumor-immune system interplay has been ushered in, leading in large part by the discovery of immune checkpoints as promising targets for successful cancer immunotherapy [2–4]. Under physiological conditions, immune checkpoints are critical regulators of immune systems that preserve immune homeostasis through stimulation and inhibition of immune responses in order to dampen the immune response following successful mitigation of an infection or other threats [5, 6]. On the other hand, these molecules are responsible for tumor cell evasion in different types of cancers. These proteins, as negative modulators, express on tumors and promote the extension of cancer cells [7, 8]. Studies have found that cancer cells are capable of evading clearance by macrophages through the overexpression of anti-phagocytic surface proteins, called “don’t eat me” signals, such as CD47 [9], programmed cell death ligand (PD-L1) [10] and β2-microglobulin (B2M) associated with class I major histocompatibility complex [11]. Each of them involves Immunoreceptor Tyrosine-based Inhibition Motif (ITIM)-based macrophage signaling, which may indicate a conserved mechanism that resulting in tumors that by nature avoid macrophage surveillance and clearance. Based on this, therapies designed to target at molecular level become one of the most promising fields in cancer therapy [12, 13]. But it cannot be ignored that there exists weaker responses to anti-PD-L1/PD-1 immunotherapies in ovarian cancer and breast cancer [14], compared with those observed in other cancers [15–17], suggesting that an alternate strategy may be required to achieve wide-ranging responses.

Cluster Differentiation 24 (CD24), also known as Heat Stable Antigen (HSA), has been extensively studied in the field of immunotherapy, and as a novel molecule for molecular-targeted drug delivery and imaging [18–20]. Because the promoter sequence of CD24 was recently confirmed, epigenetic analysis of the promoter region of CD24 has not been performed and little is known concerning the epigenetic mechanism of CD24. Human CD24 gene and its allelic isoform, cluster 4 antigen, are demonstrated to locate on chromosome 6q21 via in situ hybridization [21]. More recently, emerging data indicate that CD24 have an intriguing role in tumor evasion from phagocytosis, hypothesized to act as a “don’t eat me signal”. Bakal et al. pointed out a novel role for CD24 at the interphase of the immune system and tumor cells [22]. CD24 expression may provide immediate predictive value on responsiveness to existing immunotherapies insofar as high CD24 expression may inhibit response to therapies reliant on macrophage function.

As a potential Cancer stem cells (CSCs) biomarker and potent therapeutic target, we reviewed CD24 in regard to its molecular structure and functional roles, the expression profile in several solid cancers, as well as its connection to signaling pathways and also emphasizing its role as a potential therapeutic target for ameliorating them. Also, we report on and provide insights into both the challenges and the promises of the preclinical development of agents targeting CD24, divided according to the mechanisms and the involved pathways.

## CD24 as a cancer stem cells marker for cancer

CSCs, also known as tumor‐initiating cells, are a small subset of tumor cells which behave like normal stem cells and have self-renewal and differentiation properties. This observation was supported by clinical trials which validated the CSCs association with the tumor initiation, metastasis, recurrence and the resistance [23, 24]. Targeting CSCs has become a popular goal for treating and preventing tumor invasion [25–27]. There are currently some putative stem cell markers, which are in major use for identification and isolation of CSCs from different solid tumors [28]. CD24 has been recognized as a putative CSCs marker. Copious literatures recently describing CD24 overexpressed in various types of carcinoma, which were evident in many gene profiling analyses using microarray technology [29]. Further studies have indicated that CD24 may regulate cancer cell proliferation and invasion, supporting its usefulness as an intriguing target for novel therapies in different types of cancers [30, 31].

CD24 (25–75 KDa), consists of a small protein core with 31 amino acids [32], is a mucin-like cell surface protein depending on the cell or tissue type, which may account for the functional diversity of CD24 [21, 33, 34]. Many studies display its overexpression in many human cancers but is barely detectable in healthy human tissues [29, 35]. In addition, it is suggested that CD24 is expressed at higher level in progenitor cells or metabolically active cells but lower level in terminally differentiated cells [36]. Besides, increased expression of CD24 is usually tied with a more aggressive course of cancers. A meta-analysis strongly supports the idea that CD24 acts as an important marker of malignancy and poor prognosis in several cancers [35].Overexpression of CD24 protein or CD24 positivity occurs in a large variety of human malignancies, especially in solid tumors, including esophageal squamous cell carcinoma [37], ovarian cancer [35], prostate cancer [38], small-cell and non‐small‐cell lung carcinomas [39], breast cancer [40] as well as B‐cell lymphoma [41]. Furthermore, in several cancers, CD24 overexpression was suggested to be significantly associated with shorter patient survival time [42]. According to report, down-regulation of CD24 inhibits proliferation and induces apoptosis in tumor cells, whereas increases expression of CD24 enhances tumor growth and metastasis [43].

It is remarkable that CD24 expression is not only correlated with poor prognosis but also dependent on different pathological types [44]. In pancreatic cancer, for instance, the intracellular studies indicated CD24 expression both on membrane surface and intracellular environment but inhibited the cell invasion and metastasis [45]. It is also expressed in tumor stem cells and causes the emergence of tumor resistance and promote tumor recurrence [46]. Antibody-blocking experiments indicated that anti-CD24 monoclonal antibody can inhibit the growth of human pancreatic cell lines *in vitro *[47] . Consequently, CD24 has been exploited as one of the important CSCs markers of diagnosis and prognosis for some solid cancers and evaluated for targeted therapy in more recent years [20, 48]. Since current knowledge on the epigenetic mechanism of CD24 is very limited, it became necessary to further investigate the molecular cell biology, the clinical setting as well as genetics of CD24 in relation to cancer.

## Glycosylation pattern and biological function of CD24 in cancers

To understand the diverse functions and mechanisms of CD24 in cancers, it is necessary to comprehend its molecular structure. Composed of a short peptide backbone, there is no transmembrane domain in CD24. Additionally, it is characterized by variable glycosylation pattern in different tissues, accounting for the functional diversity of CD24 [36]. Furthermore, CD24 is also a glycosylphosphatidylinositol (GPI)-anchored protein, which means that CD24 also recruits Src family protein tyrosine kinase (Ptk) through membrane rafts to participate in signal transduction [49]. All these mentioned above may account for its various interactions and roles.

To date, the functions ascribed to CD24 include protein phosphorylation, intracellular calcium mobilization [50], induction of apoptosis, localization with lipid rafts, and transcription factor activation, assayed via inactivating approaches using extracellular antibody-mediated crosslinking [21, 33]. In addition, the absence or presence of CD24 may influence membrane raft composition and thereby affect important signaling pathways within the cells. Experimental studies during the last decade have provided evidences for the roles of CD24 overexpression in tumor anchorage-independent proliferation, migration, invasion, and apoptosis inhibition of cancer cells, as well as adhesive function during hematogenous dissemination of cancer cells [33, 51].

However, several studies conversely suggested that the absence or low expression of CD24 might be also related to tumor growth, invasiveness or metastasis in breast cancer. Furthermore, the clinicopathologic significance of CD24 is reported to be different even among the studies using the same type of carcinomas arising in the same organ. It's worthy to be claimed that membranous and cytoplasmatic CD24 have different biological activities, and therefore comprise two independent prognostic markers [20]. Further studies will be accordingly essential to demonstrate the various functions of CD24 in cancer pathogenesis and potential molecular mechanisms involved.

## Potential mechanisms of CD24 involved in cancer development

### Ligand mediated mechanisms of CD24

**Fig. 1:**
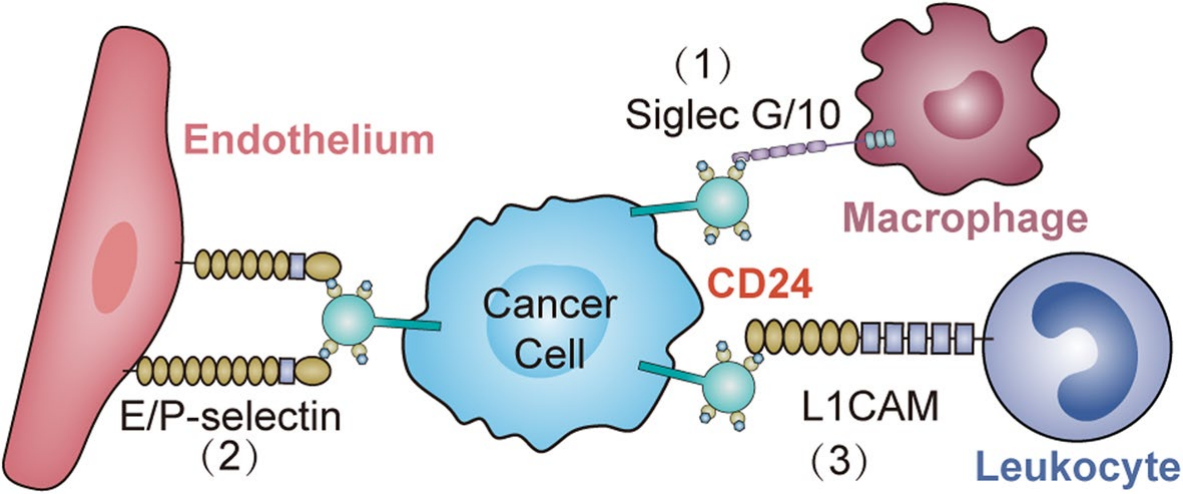
The schematic summarizes known interactions between CD24 on tumor cells and its associated ligands on immune cells at the level of surface molecules. The interaction between CD24 on cancer cells and (1) Siglec-G/10 on macrophages to inhibit phagocytosis by macrophages, (2) P-/E-selectin on endothelium and (3) L1CAM on leukocyte results in inhibitory signal cascades, thus, tumor cells cannot be cleared

As mentioned above, due to the absence of cytoplasmic domains, as well as its glycosylation is highly variable and cell type-specific, CD24 can only transduce intracellular signals by binding with protein ligands and adhesion molecules to perform a variety of functions in different tissues [52]. Several protein ligands and binding molecules were hence identified over the years (Fig. 1).

As a surface molecule expressed by activated endothelial cells and platelets, P-selectin (CD62P) is one of the most extensively studied ligand binding with CD24 [53]. It was identified that sialyl-Lewis x (SLex) carbohydrate is required for CD24-mediated rolling on P-selectin, prompting the migration and dissemination of CD24-expressing tumor cells [54]. In addition, P-selectin in the tumor microenvironment was also found to be crucial for tumor development [55]. E-selectin (CD62E) was also identified to be another CD24 ligand by Myung et al. They provided robust evidence that CD24 can influence metastasis of tumor cells by interaction with E-selectin and rearrangement actin filaments, suggesting that CD24 may have effects on adhesion complexes present on cell surface.

Another known cell membrane ligand for CD24 is L1 cell adhesion molecule (L1CAM), also known as CD171, which is recognized as another cell membrane ligand for CD24 [56]. Recently, Barash et al. discovered that heparinase has an effect on tumor progression by enhancing CD24 expression through interacting with CD24-L1CAM axis physically in glioma. CD24 binding to fibronectin type III domain of L1 thorough α 2, 3- sialic acid and also to TAG-1(Transient Axonal Glycoprotein 1) and contactin via Lewis(x) likely leads to neurite outgrowth.

In humans, to negatively regulate the immune response to proteins released by damaged cells, purified CD24 derived from tumor cell lines partners with Siglec-5 [57], whereas CD24 isolated from human placenta clearly binds to Siglec-10, and only CD24-Signlec-10 is related to phagocytosis function [58]. Inhibiting the interaction of CD24-Siglec-10 promotes the phagocytosis of CD24 + tumor cells by TAMs, and has been shown to increase survival in preclinical mouse models [22]. In addition, Siglec-10 has been reported to recognize both protein and sialic acid ligands, and thus likely has varied ligands extending beyond CD24 [22].

A physical interaction of CD24 with members of the family of Src-kinases, most likely mediated by lipid-rafts [59]. The interaction was also confirmed by Zarn et al. in lung cancer cells, which was reported that the association of the GPI-anchored CD24 and members of the Src-family of kinases most likely due to their association in lipid-rafts [60]. Another evidence supporting that Src as a CD24-mediator comes from a recent study showing that CD24 interacts with and promotes the activity of c-Src within lipid-rafts in breast cancer cells [61]. Also, it has been shown that myristylation of Src-kinase and also lipid raft integrity are essential for Src activation [62].

Therefore, because of cancer related interactions between CD24 and its identified ligands, it seems that inhibition of the interactions may be useful in anti-cancer therapies.

### Linking CD24 with signaling pathways

**Fig. 2:**
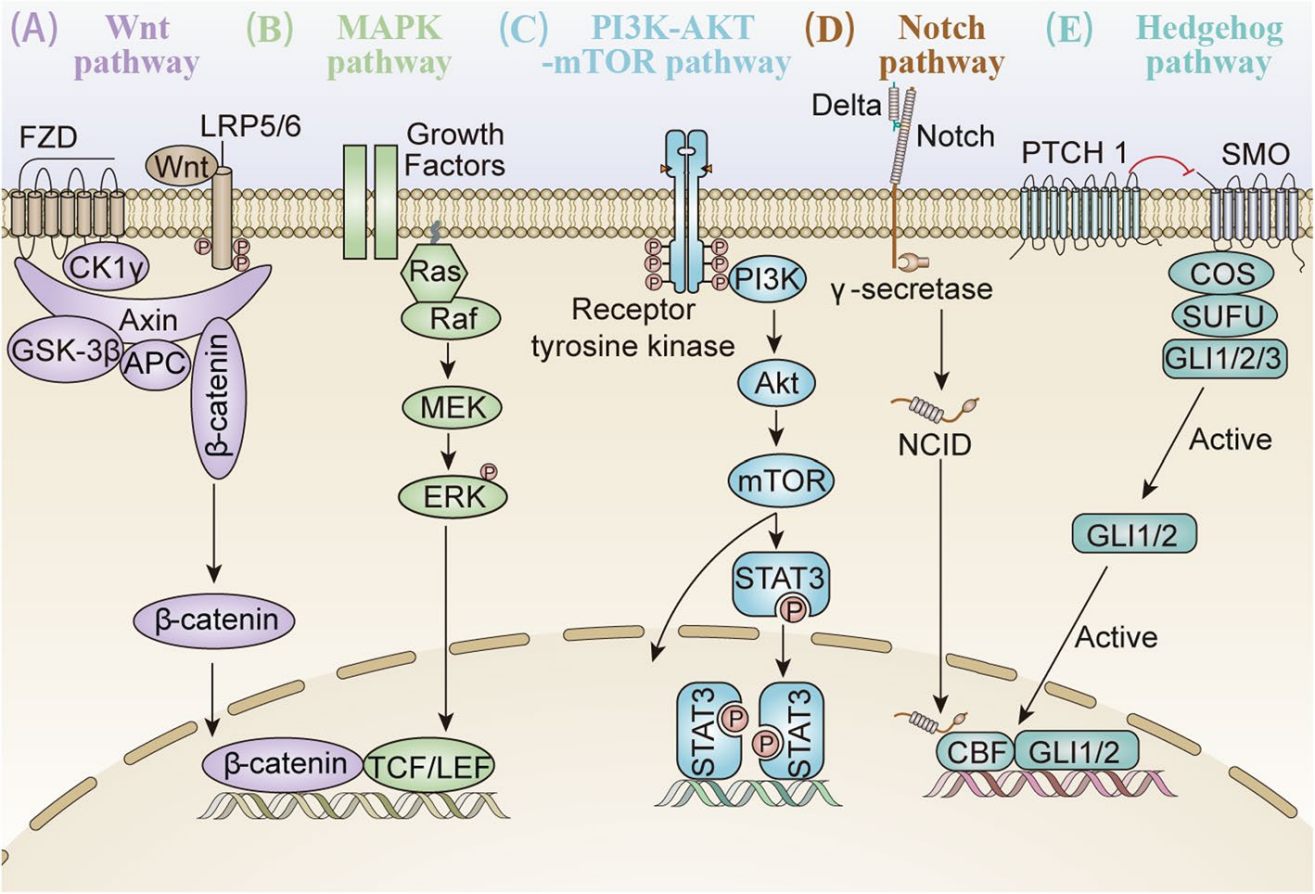
Schematic representation of the main CD24-mediated signaling pathways, Wnt/β-catenin, MAPK, PI3K/Akt/mTOR, Notch and Hedgehog. **A** The canonical Wnt signaling pathway is initiated when Wnt binds to a cell-surface receptor complex, consisting of the seven-pass transmembrane FZD and the single-pass LRP 5or 6. This leads to disassociation of the β-catenin degradation complex APC/Axin/GSK-3β, as well as β-catenin stabilization. Subsequently, the accumulated β-catenin is translocated into the nucleus, where it stimulates the transcription of TCF/LEF target genes. **B** Ras combines with Raf and mobilizes the inactive protein from the cytoplasm recruiting the Raf kinases to the plasma membrane. Once the Ras-Raf complex is translocated to the cell membrane, Ras activates the serine/threonine kinase function of Raf isoforms. Numerous effectors signals converge on RAF, activated RAF phosphorylates MEK in the cytoplasm, which in turn phosphorylates ERKs that translocate to the nucleus where they phosphorylate and regulate various nuclear and cytoplasmic substrates involved in diverse cellular responses. **C** The PI3K/Akt pathway is the upstream of mTOR, and the activation of Akt phosphorylates corresponding enzymes and kinases. In addition, mTOR regulates the promoter of downstream gene through STAT3 phosphorylation. **D** The transmembrane receptor Notch interacts with ligand Delta. Then Notch receptors are cleaved by γ-secretase, releasing NCID and subsequently activating Notch signaling. NCID is then translocated to the nucleus where it induces gene transcription by interacting with other co-factors. **E** The Hh ligands inhibit Hh pathway after binding to the twelve transmembrane protein Ptch1, which in turns interacts with the seven transmembrane protein Smo that releases five-zinc finger transcription factor GLI1/2 from a large protein complex. Abbreviations: LRP: low-density lipoprotein; FZD: frizzled; NCID: Notch intracellular domains; Hh, Hedgehog; Ptch1: Patched1; STAT3, signal transducer and activator of transcription 3; PI3K, phosphatidylinositol-3-kinase; MAPK, mitogen-activated protein kinase; Smo: Smoothened

Antibody-mediated crosslinking experiments revealed that CD24 itself has signaling capacity although it lacks an intracellular domain. Existing evidences demonstrated that CD24-mediated intracellular critical signaling was associated with protein phosphorylation, intracellular calcium mobilization, and transcription factor activation. In addition, the absence or presence of CD24 may influence membrane raft composition and thereby affect transcription factors and signaling pathways to play a critical role in the human body’s ability to ward off cancer cells [29, 63]. Signals coming from cell membrane receptors must be communicated to downstream effector molecules. So far, multiple cancer‐related signaling pathways were shown to be activated in response to changes in CD24 expression, directly or indirectly, including Wnt/β-catenin [64, 65], Mitogen activated kinase (MAPK) [66], Src or PI3K/Akt kinase [67], as well as Notch and Hedgehog pathway [68, 69], which have been analyzed in various types of cancers (Fig. 2), as described below.

#### Wnt/β-catenin signaling pathway

The Wnt signaling pathway (including both canonical and non-canonical networks) has also been associated with tumor oncogenesis including CSCs recruitment, propagation, and cross-talk [70–72]. Specifically, crosstalk between CD24 and Wnt signaling was demonstrated by Ahmad et al*.* whereby the found that CD24 may activate β-catenin to interact with the Wnt pathway and induce β-catenin translocation into the nucleus [73]. Besides, it has been shown in breast cancer that β-catenin can inhibit tumor immune escape by down-regulating the expression of CD24 [74]. And CD24 is identified as the transcriptional target of Wnt signaling in a non-transformed human mammary epithelial cell line MCF-10A [75]. Also, it has been shown that Notch and Wnt/β-catenin signaling pathways play important roles in the activation of liver cancer stem cells expressing CD24 [76]. Therefore, CD24 can exert tumorigenic properties of these cells through the activation of Wnt signaling.

#### Mitogen-Activated Protein Kinases (MAPK) signaling pathway

In response to a wide range of extracellular stimuli, MAPK cascades are shown to be involved in CD24-induced tumorigenesis in various of tumor cells. Especially, CD24 has been shown to influence the phosphorylation and activity of key components in B cells and cancer cells [77]. Also, Agrawal et al. demonstrated that CD24 expression is negatively associated with phosphorylated MAPK in cholangiocarcinoma [78].

It is worth noting that the study by Wang et al. indicated that CD24-induced cell proliferation was not only p38 MAPK-dependent but also ERK1⁄2-dependent. The study demonstrated that the ERK1⁄2 pathway was activated in colorectal cancer (CRC) and the MEK1⁄2 inhibitor abrogated CD24-induced proliferation [79]. Furthermore, the proliferation was accompanied by the elevation of activity of Raf-1 (the upstream ERK1⁄2 activator), ERK1⁄2 and p38 MAPK. In CSCs, activation of MAPK pathway leads to the promotion of cancer stem cell-like phenotypes and sustaining tumorigenicity. Therefore, CD24 indirectly mediates the maintaining of these cells by the activation of MAPK pathway, suggesting that the linkage of CD24 and this pathway may unravel a novel mechanism in the regulation of CRC proliferation.

#### PI3K/Akt mTOR signaling pathway

Except for MAPK pathway, it has also been suggested that CD24 affects the epithelial-mesenchymal transition (EMT) signal cascade via PI3K/Akt mTOR signaling pathway [80]. In addition, the PI3K/Akt pathway is the upstream of mTOR and except mTOR, and Akt also regulates a broad range of target proteins [81]. It has been shown that the CD24‑induced proliferation and invasiveness in ovarian cancer were dependent on the activation of PI3K/Akt, NF-κB and ERK [80]. Furthermore, Chen et al. indicated that colon CSCs (CD133/CD44/CD24) possess high PI3K/Akt/mTOR activities that can be inhibited by the dual inhibitor BEZ235 to achieve an effective reversal of insulin-regulated colon CSC stemness resulting in the secession of cancer cell proliferation and survival.

Recently, Bretz et al. reported that CD24 affects STAT3 phosphorylation and alters the expression of STAT3 dependent genes thorough the activation of Src [82]. In addition, inhibition of CD24 inhibited tumor growth, which was also the case for treatments targeting mTOR and the PI3K/Akt pathway and STAT3 [68, 83]. Therefore, mTOR, STAT3, and CD24 were thought to represent promising and validated targets for the inhibition of cancer growth.

#### Notch signaling pathway

Notch signaling is known to be one of the key pathways regulating tumor-initiating cells, and thus mainly targets genes that lead to high proliferation and apoptosis inhibition in cancer cells [84]. Recent studies reported that the activation of Notch signaling is necessary for maintenance of CD44^+^/CD24^−/low^ tumor-initiating cells in breast cancer [84, 85]. Besides, knockdown of CD24 led to decreased expression of Notch1, to reduction in cancer stem cell properties as well as epithelial to mesenchymal transition [86]. Moreover, Azzam et al. demonstrated that CD24^+^ cells are important for Notch1 intracellular domain signaling and metastasis formation in the therapeutically challenging triple negative breast cancer cells.

Using immunohistochemical analysis, Wan et al. also found that Notch1 expression positively correlated with CD24 expression in hepatocellular carcinoma cells (HCC) and adjacent samples, and the interactions between CD24 and the Notch1-related signaling pathways might constitute a potential regulation mechanism that is involved in HCC pathogenesis [87]. Therefore, CD24 can exert tumorigenic properties of these cells through the activation of Notch signaling.

#### Hedgehog signaling pathway

Activation of the Hedgehog (Hh) signaling pathway has been shown to result in the generation of carcinomas in vitro or in transgenic models. This pathway activates glioma-associated oncogene homolog 1 (Gli1)- and Ptch1-positive modulators of the hedgehog pathway, thereby leading to CSC proliferation. After binding to the CD24 promoter, a splice variant of Gli1 has been shown to upregulate CD24 and promote glioma invasion [88]. Many researchers have recently shown a relationship between the CD24^−/low^CD44^+^ population and Hh signaling [89, 90]. Zeng et al. discovered that the adhesion molecule CD24 may be the downstream target gene of Hh signaling using a bioinformatics approach in their previous study. Subsequently, the same authors confirmed that blocking the Hh signaling pathway was associated with significantly downregulated CD24 expression in vitro [91]. In addition, CD24 can also influence hedgehog signaling by inhibiting STAT1, resulting in the downregulation of SHH (sonic hedgehog) transcription in breast cancer cells [92].

Therefore, investigators present a novel molecular mechanism responsible for Hh signaling-mediated cancer cell migration and invasion via CD24 expression, which may be useful for the treatment of solid cancers.

#### Non-canonical CD24-mediated signaling pathway

In addition to the pathways mentioned above, there are also several novel pathways participating in performing its functions. For example, CD24 was also implied to be a downstream target of Ral GTPase signaling, involving in cancer cell motility. Moreover, CD24 and TGF-β3 interaction exhibits reciprocal regulation in human bone marrow-derived stromal cells (hBMSCs). Furthermore, the association between HER2 and CD24 is complicated, yet it is not clear which one is in the upstream of the involved signaling pathway. However, it seems that the association might be through NF-κB signaling, which is involved in CD24 and HER2 expression [93].

Therefore, inhibiting these CD24-related upstream molecules of regulatory signaling pathways can effectively prevent tumor invasion and immune escape, regulate tumor microenvironment, and thereby exert a positive effect on regressing tumor progression.

### CD24 and chemotherapy resistance

Drug resistance, especially chemotherapy resistance remains to be the critical limiting factor in cancer treatment, in which CD24 may takes part [21, 34]. Notably loss of CD24 expression was always associated with de‑differentiation, which links to invasion potential and metastasis and promotes resistance to a wide spectrum of chemotherapy drugs [94]. Recently, it has indicated that the rapid translocation of CD24 from cytosol to cell membrane was the triggering event for the acquisition of chemoresistance [34]. It is, however, important to note that membranous and cytoplasmatic CD24 have different biological activities, and therefore comprise two independent prognostic markers.

According to the existing researches, relative mechanisms involved in various survival pathways, such as HER2-Akt, ATM/NDRG2, MAPK, mTOR/Akt, Wnt/β‑catenin and STAT3 signaling pathways [95–97]. Recently, it was observed that breast CSCs are relatively resistant to chemotherapy and this resistance has been observed for CD24^‑/low^/CD44^+^ M CSCs [27, 96, 98]. Zhang et al. demonstrated that CD24 plays a role in trastuzumab-resistant HER2-expressing breast tumors through excessive activation of Src [99], suggesting that CD24 dependent activation of Src links to chemoresistance. In addition, activation of the HER2-Akt pathway by CD24 may result in lapatinib resistance [100]. Furthermore, the cytoplasmatic expression of CD24 is associated with poor overall survival in ovarian cancer, thereby CD24^−^CD44^+^ cell population is a potential indicator on drug resistance [101].

Recently, drug resistance has also been associated with the stemness of cancer cells, and the acquisition of stemness contributes to drug resistance to chemotherapy since reduction of stemness results in increased sensitivity to anti-tumor drugs [81]. Therefore, understanding the mechanisms underlying cancer stemness, especially CD24 and chemotherapy resistance is important for providing insights into the development of effective and prospective therapeutic strategies against solid cancers.

## Regulation of CD24 expression in solid caners

### Ovarian cancer

The CD24 expression and its significance in Ovarian cancer (OC) tumors have been demonstrated in several studies. Kristiansen et al*.* observed that CD24 commonly expressed (cytoplasmic; 59% and membranous; 84%) in invasive ovarian carcinomas whereas there was no expression on surface epithelium of normal ovaries as well as ovarian adenomas [102]. Therefore, CD24 expression has been used as an independent prognostic marker of survival in patients with OC. In addition, Moulla et al*.* also noticed during tumor progression cytoplasmic expression of CD24 increased, which may be due to overproduction of proteins and also disturbance of their distribution or degradation within the malignant cell [103].

Recently, it was found that Ovarian cancer chemoresistance and recurrence are possibly caused by the presence of residual cancer cells that have CSCs [104]. Gao et al*.* isolated a subset of CD24^+^ cells from ovarian tumor specimens of a patient that possess CSCs properties [105]. Then, the function of CD24 in CSCs was investigated by Burgos‐Ojeda *et al *[106] , who investigated the phosphorylation of STAT3 was related to activation of JAK2 kinase in CD24-expressing cells. However, further in vitro and in vivo investigations are needed for identification of the exact mechanism of CD24 in OC cells.

### Breast cancer

Certain previous studies have reported that positive CD24 expression increases the metastatic potential of malignant cells and is associated with poor clinical outcomes in breast carcinomas. A pioneering study indicated that CD44 + /CD24- cells can be recognized as prospective breast cancer stem cells [63]. In addition, CD24 was also revealed to be involved in the regulation of stemness and the epithelial to mesenchymal transition in breast cancer cells [107]. Lastly, Young et al*.* demonstrated that CD24 is epigenetically regulated in association with histone modification in breast cancer cells [107], but little is known regarding the mechanism underlying the transcriptional regulation of CD24.

Remarkably, comparing with luminal and HER2 phenotypes, triple negative breast cancer (TNBC) has abundance of CD44 + /CD24- stem cells, due to their potent self-renewal and differentiation capacities [108, 109]. However, the majority of clinical studies ignore the impact of the treatments on these specific subpopulations. Therefore, researchers should further characterize CD24 function in the tumor biology of different phenotypes for better understanding of the mechanisms and developing novel, effective targeted therapies to improve outcome of breast cancer patients.

### Lung cancer

A series of findings on CD24 included prominent expression in tumor epithelium, association with pro-tumor immune phenotypes and reduced survival, and functional role in vivo. CD24 was reported by Sinjab et al. to be at the core of an enriched cell–cell interactome in early-stage lung adenocarcinoma (LUAD), which is associated with a pro-tumor immune contexture and poor prognosis as well as promotes LUAD growth in vivo [110]. Furthermore, Ozawa et al. demonstrated that CD24 was a candidate negative predictive marker of immune-checkpoint inhibitors (ICI) in advanced, non-small-cell lung cancer (NSCLC) with PD-L1 TPS < 50 [111], which suggested that expression of CD24 was associated with changes in factors related to monocytes and angiogenesis after ICI initiation. Thus, CD24 may be a potential marker of invasion and prognosis in lung cancer, and as well as a viable target for treatment of early-stage lung cancer [110]. Additionally, QIAO et al. found that CD24 and molecular chaperone heat shock protein 70 (Hsp70) were highly expressed in lung cancer tissues, and associated with invasion, metastasis, and poor survival [112]. Co-expression of CD24 and Hsp70 may be a prognostic biomarker for lung cancer. And Hsp70 may be regulated by CD24 expression.

Remarkably, Xu et al. in their report found that CD24 didn’t exert any stronger tumor formation ability in vivo, which is the gold standard of CSCs. Therefore, future studies are needed for larger-sample studies to answer the question of whether CD24 can be considered as all kinds of lung CSCs marker.

### Colorectal Cancer (CRC)

CD24 has been found in 90% of adenoma and 86% of malignant lesions, which suggests that the change in the expression of CD24 is involved in the occurrence and development of colorectal cancer (CRC) [61, 113]. A series of studies have indicated that CD24 increased with age, the diameter of colorectal polyps, the type of dysplasia of colorectal polyps, the metastasis of CRC, and the degree of differentiation of CRC, which showed a significant positive correlation [114, 115]. Moreover, Huang et al. demonstrated that patients with CD133-high/CD24-low tumors have worse Disease specific survival (DSS) and overall survival (OS), and are more likely to have early and late recurrences [64]. Thus, some studies have shown that CD24 has a great relationship with CRC treatment and can be used as a target for targeted therapy of CRC.

In a recent report, downregulation of CD24 in CRC by RNA interference or anti-CD24 monoclonal antibodies significantly inhibited tumor development in vitro and in vivo [116]. Furthermore, due to decreased proliferation and absence of stemness features associated with CD133-high/CD24-low tumors, the combination of CD133-high/CD24-low characterization is associated with poorer prognosis and greater recurrence [64].

## Anti-CD24 antibody monotherapy and the clinical trials for cancer therapies targeting CD24

While as a CSCs marker and as a therapeutic target, CD24 remains incompletely understood, the value of targeting it in tumor treatment has been increasingly confirmed. Studies suggested that the CD24-targeted antibody could offer robust cancer cell-killing effects and block the vicious cycle for tumor recurrence from CSCs. In the world, several active oncology clinical trials were registered on ClinicalTrials.gov to evaluate the efficacy of anti-CD24 based tumor therapy in preclinical models at the time of manuscript writing. Here, we reviewed and summarized recent advances in anti-CD24 antibodies therapy were evaluated in seven studies on cancer patients (Table 1).

**Table 1:** Clinical trials targeting CD24 registered with the National Clinical Trials Registry (NCT) system

NCT ID	Phase	Treatment	Malignancy type	Enrollment	Endpoints	Locations	Current Status
NCT04060407	1b/2	CINDI	Metastatic Melanoma	0	Business Reasons	Huntsman Cancer Institute, Salt Lake City, Utah, USA	Withdrawn
NCT04552704	1/2	TIRAEC	Advanced Malignant Solid Neoplasm	3	Sponsor Change	University of California Davis Comprehensive Cancer Center Sacramento, California, USA	Terminated
NCT04095858	3	CD24Fc/tacrolimus / methotrexate (CD24Fc/Tac/MTX)	Hematopoietic Stem Cell Transplantation Acute Graft Versus Host Disease Acute Myeloid Leukemia Acute Lymphoblastic Leukemia Myelodysplastic Syndromes	11	Business Reasons	City of Hope (Site 0302), Duarte, California, USA The University of Chicago Medical Center (Site 0306), Chicago, Illinois, USA Penn State University Milton S. Hershey Medical Center (Site 0304), Hershey, Pennsylvania, USA Abramson Cancer Center of the University of Pennsylvania (Site 0309), Philadelphia, Pennsylvania, USA	Terminated
NCT02663622	2	Phase II Trial of CD24Fc for the Prevention of Acute GVHD Following Myeloablative Allogeneic HSCT (MK-7110–002)	Graft Versus Host Disease Hematopoietic Stem Cell Transplantation Leukemia	44		Indiana University School of Medicine, Indianapolis, Indiana, USA The University of Michigan Comprehensive Cancer Center, Ann Arbor, Michigan, USA Karmanos Cancer Institute, Detroit, Michigan, USA Ohio State University, Columbus, Ohio, USA	Completed
NCT01214512	Not Provided	Performance Evaluation of the Micromedic CD24 in Vitro Diagnostic Assay	Colorectal Cancer	229	Not Provided	Bat Yamon Gastroentrology Clinic, Bat Yam, Israel Zamenhoff Gastroentrology Clinic, Tel Aviv, Israel	Completed
NCT01265225	Not Provided	Prognostic Value of Stem Cell Related Markers	Breast Cancer	0	No Finance	Baruch PMC, Poriya, Lower galilee, Israel	Withdrawn
NCT04907422	Not Provided	Diagnostic and Prognostic Accuracy of Gold Nanoparticles in Salivary Gland Tumours	Carcinoma Ex Pleomorphic Adenoma of Salivary Glands Pleomorphic Adenoma of Salivary Glands	60	Not Provided	Faculty of Dentistry, October 6 University, Giza, Egypt	Completed

*Abbreviations*: *CINDI* CD24Fc With Ipilimumab and Nivolumab to Decrease irAE, *TIRAEC* Treatment of Immune Related Adverse Events in Patients with CD24Fc, *GVHD* Graft Versus Host Disease

In the U.S., a phase 1b/II trial is underway to evaluating the safety and efficacy of combining CD24Fc with ipilimumab and nivolumab to reduce the toxicity of immunotherapy combination, in patients with Metastatic Melanoma who are naïve to anti-PD1/L1 based checkpoint inhibitors. However, this study was withdrawn on 1 June 2021 for business reasons (ClinicalTrials.gov Identifier: NCT04060407).

The second phase I/II trial is investigating the side effects and how well CD24Fc works in treating immune related adverse events in patients with Advanced Malignant Solid Neoplasm that have spread to other places in the body (advanced). Researchers expect that adding CD24Fc (CD24 Extracellular Domain-IgG1 Fc Domain Recombinant Fusion Protein CD24Fc) to standard treatment may shorten the recovery time and reduce the severity of side effects from immunotherapy. However, this study was terminated early due to the sponsor changed (3 out of 6 patients enrolled) in phase I study on 3 February 2021 (ClinicalTrials.gov Identifier: NCT04552704).

The third phase III trial is underway to test CD24Fc for the prevention of Acute Graft Versus Host Disease (GVHD) following myeloablative Hematopoietic Stem Cell Transplantation (HSCT) (MK-7110–005) (CATHY). The study compares two acute graft-versus-host disease (aGVHD) prophylaxis regimens: CD24Fc/tacrolimus/ methotrexate (CD24Fc/Tac/MTX) versus placebo/tacrolimus/methotrexate (placebo/Tac/MTX) in the setting of myeloablative conditioning (MAC), matched unrelated donor (MUD) allogeneic hematopoietic stem cell transplantation in 11 participants with acute leukemia (AML/ALL) or myelodysplastic syndrome (MDS). Unfortunately, this study was also terminated on 5 November, 2021 for business reasons (ClinicalTrials.gov Identifier: NCT04095858).

The fourth trial is fairly similar to the previous one; 44 patients with acute graft versus host disease (GvHD), hematopoietic stem cell transplantation (HSCT) or acute leukemia. Patients were divided into four groups, each receiving a different treatment dosage (Placebo, dose 1: CD24Fc 240 mg arm, dose 2: CD24Fc 480 mg arms or dose 3: CD24Fc 960 mg arm). This is a multicenter prospective phase IIa dose escalation and phase IIa expansion cohort clinical trial designed to evaluate the safety and tolerability of CD24Fc for acute GvHD prophylaxis. The actual study completion date is 18 May 2021 (ClinicalTrials.gov Identifier: NCT02663622).

In Israel, a completed trial is enrolling 229 patients with colorectal cancer, aged 50 years and above that were referred to colonoscopy assessment. This study was aimed to evaluate the performance of the Micromedic CD24 assay in identifying colorectal adenoma using Western blot and ELISA assays. These assays are designed to detect CD24 protein in peripheral blood leukocytes (PBL). Elevated levels of CD24 may be indicative of colorectal adenoma. The study completion date is in August 2012 (ClinicalTrials.gov Identifier: NCT01214512). A second trial is underway to evaluate prognostic value of stem cell related markers (CD24, CD44, CD326 and EPCR) for predicting breast cancer recurrence in tumor stages 0-II, but it was withdrawn for lacking of financial support (ClinicalTrials.gov Identifier: NCT01265225).

In Egypt, an observational clinical trial is completed, enrolling 60 patients with Carcinoma Ex Pleomorphic Adenoma of Salivary Glands [117]. To identify the most sensitive and specific diagnostic biomarker to be used in detecting salivary gland tumors, the study has critically introduced a detection strategy based on nanoparticles for salivary gland tumors, and evaluated diagnostic potential of CD24-AuNC (index test) compared to non-conjugated CD24 (reference test) in determination of salivary gland tumors. And this current study concluded that CD24-AuNC provide a promising sensitive and specific diagnostic and prognostic tool for CSCs identification in diagnostically challenging SG tumors. The actual study completion date is 3 February 2021, and the results have yet to be published (ClinicalTrials.gov Identifier: NCT04907422).

## Anti-CD24 antibody monotherapy for COVID-19 therapy

It's worth mentioning that the world has changed with the fast expansion of the Coronavirus disease (COVID-19). Impaired immune cell function leading to prolonged uncontrolled inflammation is the hallmark of severe COVID-19 pathology [118]. At present, many treatment options have been employed to overcome it. In recent studies, CD24 has attracted attention as a promising strategy for treating COVID-19 due to its known anti-inflammatory action, especially soluble CD24 may reduce COVID-19-associated systemic immunopathology [119]. There are several trials targeting CD24 are currently ongoing in patients with moderate or severe COVID-19 infection around the world.

The first study is a phase I study (NCT04747574), called ‘‘EXO-CD24″, which is a biologic therapeutic agent that works inhibiting the cytokine storm. The exosomes, used as a vehicle, deliver CD24 directly to the target organ [120]. Besides, the exosomes used in the study were isolated from T-RExTM-293 cells that overexpressed CD24. Importantly, CD24Fc and CD24 exosomes may act through Siglec stimulation to protect against severe COVID-19. The treatment achieved in its Phase I tests more than 95% of effectiveness, helping 29 of 30 patients to quickly recover from the disease. COVID-19 patients with a moderate or severe condition were treated between three to seven days. Therefore, the direct administration route throughout an inhalation device opens new possibilities for the use of engineered exosomes as a new therapy for lung cancer treatment.

The second is a phase II study, composed of 155 patients with moderate or severe COVID-19 infection, to evaluate the safety and efficacy of exosomes overexpressing CD24 to prevent clinical deterioration. The exosomes will be isolated and purified from human embryonic kidney T-REx™-293 cells that constitutively express high levels of human CD24. The estimated primary completion date is scheduled for July 2022 ((ClinicalTrials.gov accessed on 22 August 2022) Identifier: NCT04969172).

The third trial, is fairly similar to the previous one; 90 patients with moderate or severe COVID-19 infection will be divided into two cohorts (dose 1: 10^9 exosome particles (per dose) versus dose 2: 10^10 exosome particles (per dose)). The aim of this phase II study is to evaluate the safety and efficacy of exosomes overexpressing CD24 of two doses. The estimated primary completion date is scheduled for September 2021, but is still recruiting ((ClinicalTrials.gov accessed on 22 August 2021) Identifier: NCT04902183).

The fourth trial recently completed, is a double-blind phase III study (OncoImmune Inc.) to evaluate the safety and efficacy of CD24Fc (MK-7110) in 234 patients with COVID-19 infection and receiving oxygen support. CD24Fc is a protein, consisting of two molecules of CD24 attached to a single human IgG1 Fc [121]. Given in conjunction with the anti-viral drug, Remdesivir, a phase III trial will involve 234 critically ill patients randomized into blinded placebo and CD24-Fc arms, with time to clinical improvement from severe to mild symptom as the primary endpoint. The results indicated that CD24-Fc may significantly short recovery time, and reduce the disease progression and mortality, acting a specific immune modulatory role, which is superior to older generations of anti-inflammatory medications. The study was completed on 20 October 2020 ((ClinicalTrials.gov accessed on 22 August 2021) Identifier: NCT04317040).

Although the trials targeting CD24 have been clinically evaluated in COVID-19 patients with promising preliminary outcomes, their development will require additional investigation in future.

## Limitations, challenges and future prospects of targeting CD24

Due to its high expression in cancers and its role as a biomarker of some CSCs and an antiphagocytic checkpoint, CD24 emerges as a potential therapeutic target for cancer immunotherapy, and its role has been reviewed previously [21, 122]. Antibodies targeting this checkpoint are in preclinical or clinical trials. From the perspective of targeting phagocytosis, none of these drugs are on the market to date yet. Some drugs have been developed based on other immune responses, but more studies will be performed on phagocytosis checkpoint drugs.

As is well-known, the functional roles of CD24 in signaling pathways and cell communication of a small peptide with no transmembrane domain remain challenged to decipher. Additionally, targeting phagocytosis also faces other potential limitations and challenges. Firstly, the function of this phagocytosis checkpoint mainly relies on innate responses that are less specific and may induce tissue damage to normal tissues in addition to tumors, especially when phagocytosis targeting is used combing with other immune-modulating methods. Secondly, therapies targeting this signal are well known to lead to increased phagocytosis of cancer cells; however, because “don’t eat me” signals are markers of “self” on normal cells, treatment can result in negative off-target effects, such as anemia and B-cell depletion. Thirdly, not all cancers uniformly overexpress or suppress “don’t eat me” signals and significant variability may exist between malignancies and even individual patients. Fourthly, unlike hematological malignancies, solid tumors are located in specific sites (not always near blood vessels) and are frequently protected by an immune-silencing microenvironment.

Additionally, its expression on immune cells leads to harmful adverse effects, however, investigators have also attempted to explore the efficacy of anti-CD24- based cancer therapy in preclinical models. Specifically, the local application of the anti-CD24 antibodies, combined with immunotherapy such as chimeric antigen receptor (CAR)-T, tumor vaccines, immune checkpoint inhibitors, or other systemic immunotherapies seem to be most promising [123, 124]. For example, selective removal of sialic acid from tumor cells using antibody-sialidase conjugates has been verified to significantly enhance tumor cell susceptibility to ADCC and enable immune cell killing of desialylated cancer cells. Moreover, clinical trials are also imperative to confirm the safety and efficacy of currently approved drugs in global population and develop novel personalized therapies specific to different patients.

Fortunately, novel technologies developed in recent years, such as next-generation sequencing platforms endowed of high-resolution property for cellular difference detectability, or high-throughput drug screenings, as well as cancer screening and precision medicine would provide new insights of drug response of CD24 and would help define which type and stage of various cancers is most amenable to be treated with one or more specific types of anti-CD24 therapies.

## Conclusion

In conclusion, highly expressed CD24 in solid cancers appear to be both a prognostic biomarker as well as a promising therapeutic target, thus an opportunity is offered for researchers to study and use it in cancer treatment research and drug development. More critically, since the outbreak of COVID-19, CD24-Fc have also been identified to treat novel coronavirus patients, for reducing the inflammatory response caused by viruses and maintaining the stability of the immune system. More critically, it is faced with many new challenges, and further investigation of the mechanisms underlying tumor-mediated immune evasion might overcome these challenges and promote the development of the first drug targeting phagocytosis checkpoints. It is hoped that, in the near future, cancer therapy targeted against CD24 may be a promising strategy for the treatment of solid cancers metastasis and recurrence in future.

## Data Availability

All data generated or analysed during this study are included in this published article.

## Abbreviations

B2M: β2-Microglobulin
CD24: Cluster Differentiation 24
CAR: Chimeric Antigen Receptor
CSCs: Cancer stem cells
EMT: Epithelial-mesenchymal transition
GPI: Glycosylphosphatidylinositol
HAS: Heat Stable Antigen
ITIM: Immunoreceptor Tyrosine-based Inhibition Motif
L1CAM: L1 cell adhesion molecule
PD-L1: Programmed cell death ligand 1
PBL: Peripheral Blood Leukocytes
